# Dimension- and position-controlled growth of GaN microstructure arrays on graphene films for flexible device applications

**DOI:** 10.1038/s41598-021-97048-2

**Published:** 2021-09-01

**Authors:** Dongha Yoo, Keundong Lee, Youngbin Tchoe, Puspendu Guha, Asad Ali, Rajendra K. Saroj, Seokje Lee, A. B. M. Hamidul Islam, Miyoung Kim, Gyu-Chul Yi

**Affiliations:** 1 Department of Physics and Astronomy, Institute of Applied Physics, and Research Institute of Advanced Materials, Seoul National University, Seoul, 151-747 Korea; 2 Department of Materials Science and Engineering, Research Institute of Advanced Materials, Seoul National University, Seoul, 151-744 Korea

**Keywords:** Electronic properties and materials, Electronic devices

## Abstract

This paper describes the fabrication process and characteristics of dimension- and position-controlled gallium nitride (GaN) microstructure arrays grown on graphene films and their quantum structures for use in flexible light-emitting device applications. The characteristics of dimension- and position-controlled growth, which is crucial to fabricate high-performance electronic and optoelectronic devices, were investigated using scanning and transmission electron microscopes and power-dependent photoluminescence spectroscopy measurements. Among the GaN microstructures, GaN microrods exhibited excellent photoluminescence characteristics including room-temperature stimulated emission, which is especially useful for optoelectronic device applications. As one of the device applications of the position-controlled GaN microrod arrays, we fabricated light-emitting diodes (LEDs) by heteroepitaxially growing In_x_Ga_1−x_N/GaN multiple quantum wells (MQWs) and a *p*-type GaN layer on the surfaces of GaN microrods and by depositing Ti/Au and Ni/Au metal layers to prepare n-type and p-type ohmic contacts, respectively. Furthermore, the GaN microrod LED arrays were transferred onto Cu foil by using the chemical lift-off method. Even after being transferred onto the flexible Cu foil substrate, the microrod LEDs exhibited strong emission of visible blue light. The proposed method to enable the dimension- and position-controlled growth of GaN microstructures on graphene films can likely be used to fabricate other high-quality flexible inorganic semiconductor devices such as micro-LED displays with an ultrahigh resolution.

## Introduction

Recently, hybrid-dimensional heterostructures consisting of GaN microstructures with two-dimensional (2D) graphene films have attracted attention for use in transferable and flexible electronic and photonic devices^1–7^. In such heterostructures, the GaN microstructures provides a light-emitting active region through radiative recombination, which is significant in fabricating light-emitting devices and displays^8–10^. Moreover, when combined with a graphene film substrate, GaN microstructures demonstrate excellent tolerance against mechanical deformation^11,12^. Although GaN hybrid-dimensional heterostructures can be applied to prepare several types of flexible devices, the most promising device application pertains to a flexible high-resolution display with a micrometer-sized light-emitting diode (micro-LED) array^13,14^. For such applications, the dimensions and positions of the GaN microstructures must be precisely controlled for the individual operation of each micro-LED in the array. Nevertheless, the key criteria for optimizing the device performance, such as the morphology and position control, *n*- and *p*-type doping, and formation of quantum structures in the GaN microstructures, have not been extensively studied^15^. In this study, we realized the dimension- and position-controlled growth of GaN microstructures on graphene films and the formation of their heterostructures with In_x_Ga_1−x_N/GaN multiple-quantum-well structures for use in flexible micro-LED applications. The precisely controlled growth of GaN microstructure on graphene films can help develop templates for the individual operation of the devices for micro-LED displays.

## Materials and methods

**Figure 1:**
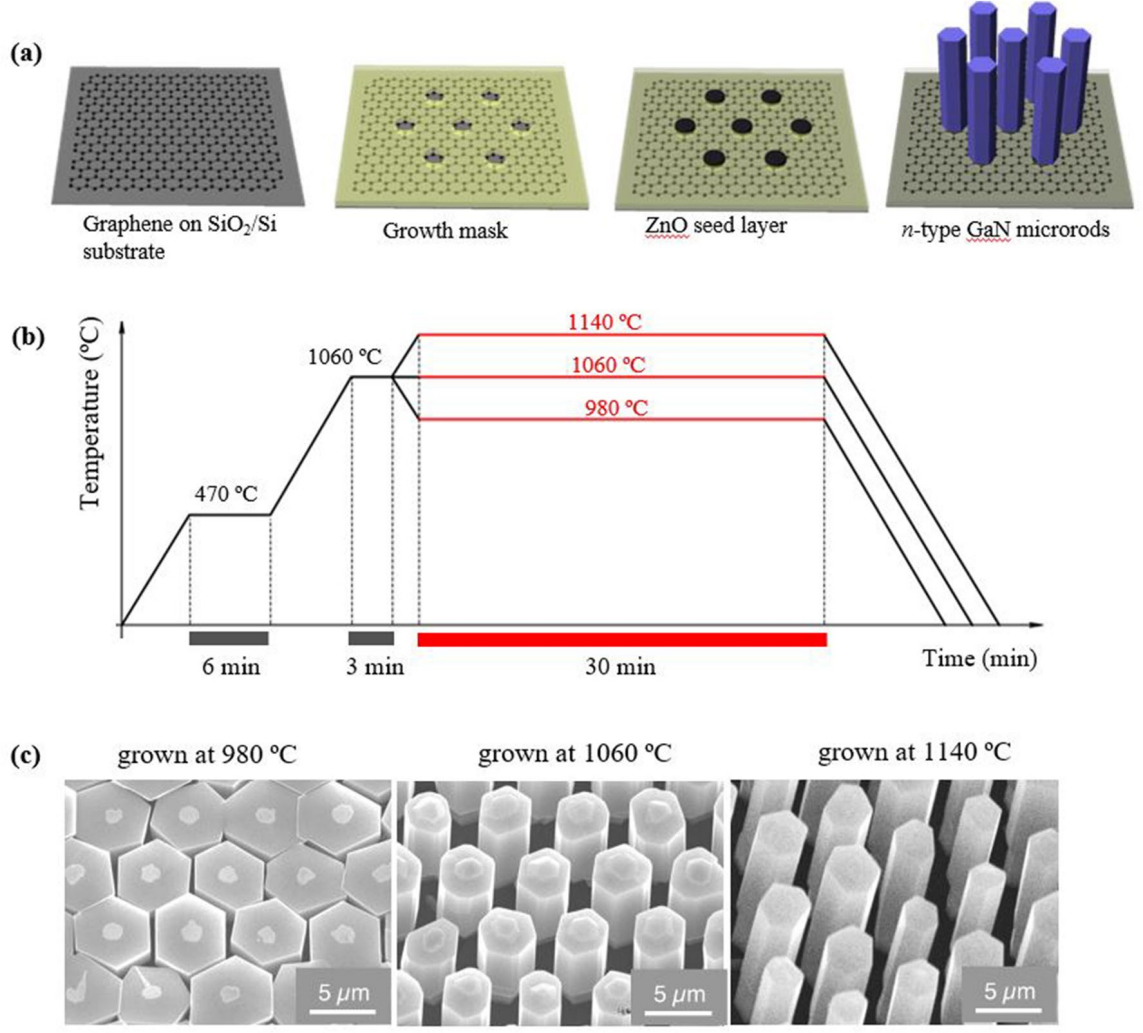
Dimension- and position-controlled growth of GaN microrod heterostructures. (**a**) Schematic illustration of GaN microrods growth on graphene films. (**b**) Graph of three-step growth method (**c**) SEM images grown at 980 °C, 1060 °C, and 1140 °C.

Our basic strategy to realize the controlled growth of GaN microstructures was to enhance nucleation and control the growth direction of the microstructures on the graphene surface. Because the lack of chemical reactivity of graphene renders it challenging to prepare dimension-controlled GaN microstructures on graphene films, we employed a ZnO seed layer to enhance the nucleation of GaN on graphene. Figure 1a schematically shows the process of growing position-controlled GaN microstructure arrays on graphene films. First, a large graphene film that was synthesized on a Cu foil by using chemical vapor deposition (CVD) system was transferred on a Si wafer with a 50-nm-thick native silicon dioxide (SiO_2_) layer. To control the positions of the microstructures in the array, a patterned SiO_2_ growth mask layer with microhole arrays having a diameter and interdistance of 1 µm and 8 µm, respectively, was fabricated on the graphene film, and the microstructures were grown only on the patterned microhole sites. Subsequently, we grew a 0.5-µm-thick ZnO seed layer before the growth of GaN microstructure arrays to enhance the nucleation. The complete ZnO seed layer was deposited on each microhole pattern array (see Figure S3, Supplementary Information). When GaN was grown on graphene without using the ZnO seed layer, all the GaN microrods were not grown on the patterned sites. As shown in supplementary Figure S1, only a few micrometer-sized islands were formed randomly, presumably due to the rare nucleation of GaN on pristine graphene^16^. This result suggests that the ZnO seed layer plays an essential role in enhancing the nucleation and realization of the vertically aligned growth of GaN microstructures on graphene films.

The position- and dimension-controlled growth of the GaN microstructures was achieved by setting suitable growth parameters. First, we used a multistep-temperature-growth method, as shown in Fig. 1b. When GaN was grown at a high temperature of 1060–1140 °C without the low-temperature of 470 °C, a few micrometer-size islands were formed randomly, presumably because the ZnO seed layer was etched out under a hydrogen environment at a high temperature, as shown in Figure S2a. Accordingly, a thin GaN film was coated on the ZnO seed layer under a nitrogen environment at a low temperature of 470 °C^17^. Next, the growth temperature was increased to high values of 980–1140 °C. Figure 1c shows the scanning electron microscope (SEM) image of the GaN microstructures grown at 1060 °C. By implementing two-step growth at 470 and 1140 °C, the GaN microstructures could be selectively grown; however, the GaN microstructure could not be epitaxially grown at excessively high temperatures (see Figure S2b). The aspect ratio of the GaN microrods was controlled through a three-step growth process. Figure 1c shows the SEM images of the GaN microstructures grown at various third-step temperatures of 980, 1060, and 1140 °C with constant first- and second-step temperatures of 470 and 1060 °C, respectively. The SEM images indicate that diameters and heights of the microstructures were 3.8 $\pm$ 0.1 and 1.9 $\pm$ 0.1, 2.9 $\pm$ 0.3 and 6.1 $\pm$ 0.3, and 1.9 $\pm$ 0.3 μm and 11.8 $\pm$ 0.6 μm for the third-step temperatures of 980, 1060, and 1140 °C, respectively. In particular, with the increase in the growth temperature, the diameters and heights of the microstructures decreased and increased, respectively. The aspect ratio of microstructures increases with increase in the growth temperature due to the different adsorption and desorption rates of the precursors at different growth temperatures^18,19^. Notably, at a high temperature, the surface diffusion of the Ga adatom from the side-wall to the top increases, and more diffused Ga adatom can react with NH_3_ on the microrod tips, resulting in the enhanced vertical growth of the microstructures.

## Results and discussion

**Figure 2:**
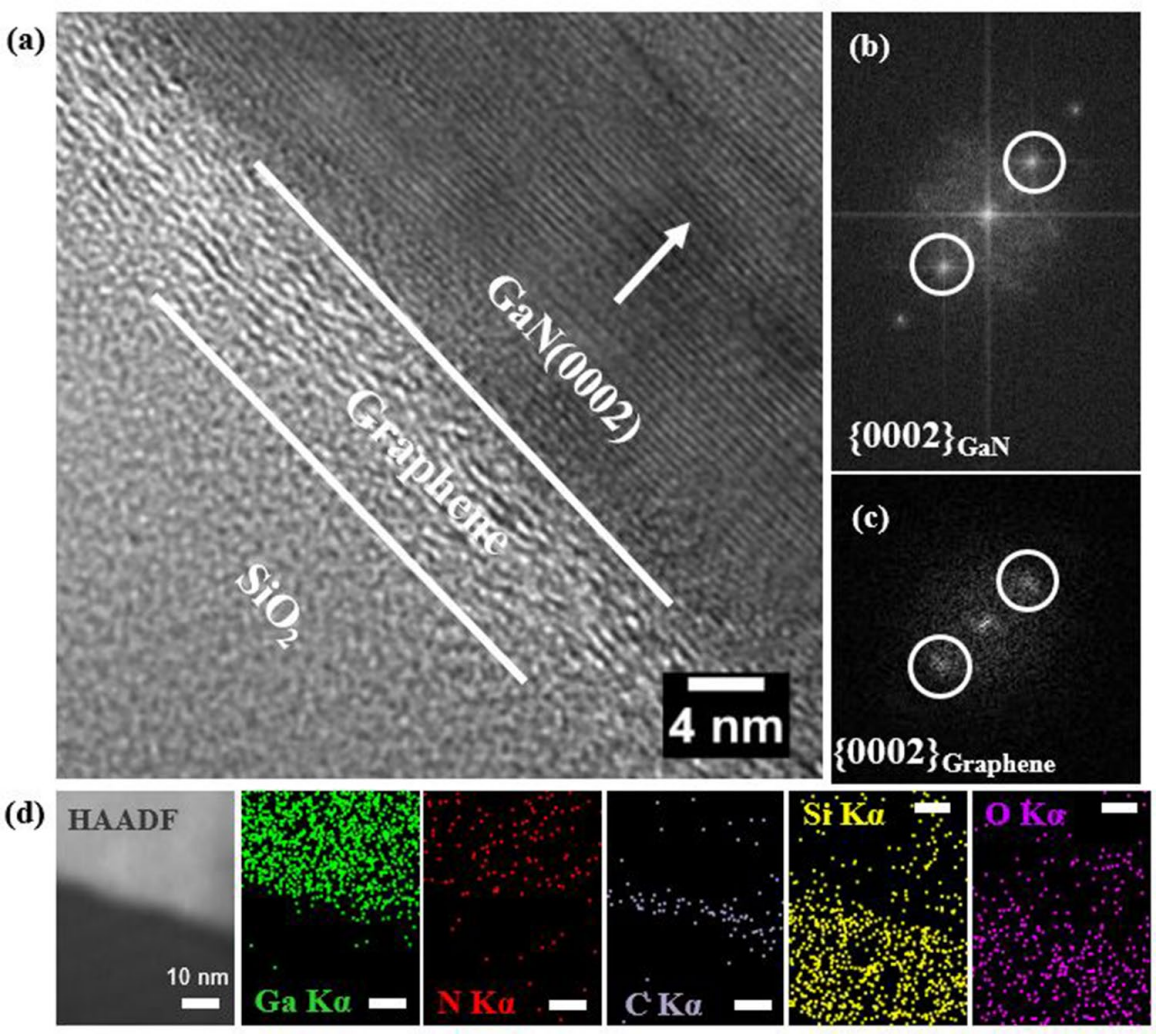
TEM of GaN microstructures on graphene layers; (**a**) Cross-sectional high-resolution (HR) TEM from the sample; (**b**) FFT pattern from GaN layers; (**c**) FFT pattern from graphene film. (**d**) STEM-EDS (cross-sectional view): HAADF image, mapping of Ga, N, C, Si and O elements.

The atomic arrangement of the GaN microstructures and heteroepitaxial interface between the GaN and graphene layers were investigated using cross-sectional high-resolution transmission electron microscopy (HR-TEM) and fast Fourier transform (FFT) techniques. Figure 2a shows the HR-TEM image of the GaN microstructures on the graphene layers. An ordered atomic structure for GaN was observed in the initial configuration of growth on the graphene layers. From the TEM image, the lattice spacing values were calculated to be 0.34 nm and 0.26 nm, corresponding to d(0002) of graphene and d(0002) of GaN, respectively. The FFT patterns pertaining to the GaN and graphene layers are presented in Fig. 2b and c, respectively. The patterns indicate that GaN was epitaxially grown on graphene films because {0002}GaN was parallel to {0002}graphene. Even taking the low-temperature GaN capping layer in Nitrogen atmosphere, most of ZnO is dissolved. These results suggest that most of the ZnO seed layers disappeared during GaN growth.

The energy-dispersive X-ray spectroscopy (EDS) maps of the interface between GaN and graphene clearly demonstrated the chemical composition of GaN microstructures grown on graphene. Figure 2d presents the high-angle annular dark-field (HAADF) image and elemental maps pertaining to the HAADF image region for Ga Kα, N Kα, C Kα, Si Kα, and O Kα in the scanning TEM (STEM) mode (STEM-HAADF). Notably, in STEM-HAADF images, a higher contrast implies a higher Z value of the constituent element in the sample. The distributions of Ga and N could be attributed to GaN; C was derived only from the graphene layers (sandwiched between GaN and SiO_2_); Si and O could be attributed to the SiO_2_ layer. The higher Ga concentration with respect to N concentration could be attributed to the focused ion beam sample preparation using a Ga ion source.

**Figure 3:**
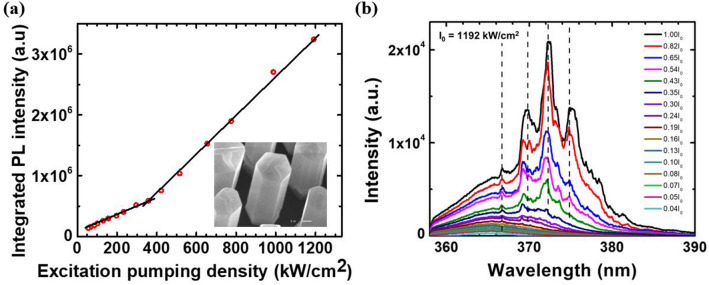
(**a**) The plot of integrated PL intensity as a function of excitation density at 350 KW/cm^2^. (**b**) Representative power-dependent PL spectra of GaN microrod.

Furthermore, the optical quality of the GaN microstructures grown on graphene was examined using power-dependent micro-photoluminescence (μ-PL) spectroscopy. For the optical characterizations, we used the hexagonal arrays of GaN microrods with a diameter of 3.9 ± 0.2 μm and height of 15.8 ± 0.4 μm (shown in the inset of Fig. 3a) and having a flat top surface and sidewalls. Figure 3a shows the room-temperature μ-PL spectra of the microrods measured at different excitation densities ranging from 60 to 1190 kW/cm^2^. At excitation densities below 350 kW/cm^2^, a wide emission peak at 367 nm was captured without any remarkable feature shown in Fig. 3b. However, as the excitation density increased to more than 350 kW/cm^2^, sharp peaks appeared at 367.4, 370.2, 372.9, and 375.8 nm with a regular spacing of 2.8 nm around the near-band-edge emission, which eventually emerged as the dominant feature in the PL spectra. Each waveguide mode underwent Fabry–Perot (FP) oscillations, thereby generating a number of resonance peaks in the lasing spectrum^20^. The FP resonance of the mode spacing was defined as ∆λ = λ2/2L[1/(n-λdn/dλ)], where ∆λ is the mode spacing, L is the cavity length, n is the effective refractive index, and dn/dλ is the first-order dispersion of the effective refractive index^21^. ∆λ was calculated to be 2.83 nm, which was consistent with the measured ∆λ. The observation of the lasing characteristics with a low threshold pumping density indicated that the GaN microrods grown on graphene had a high optical quality. It was considered that the GaN microrods having flat side facets, which were naturally grown without etching, enhanced the lasing properties by lowering the diffraction loss.

**Figure 4:**
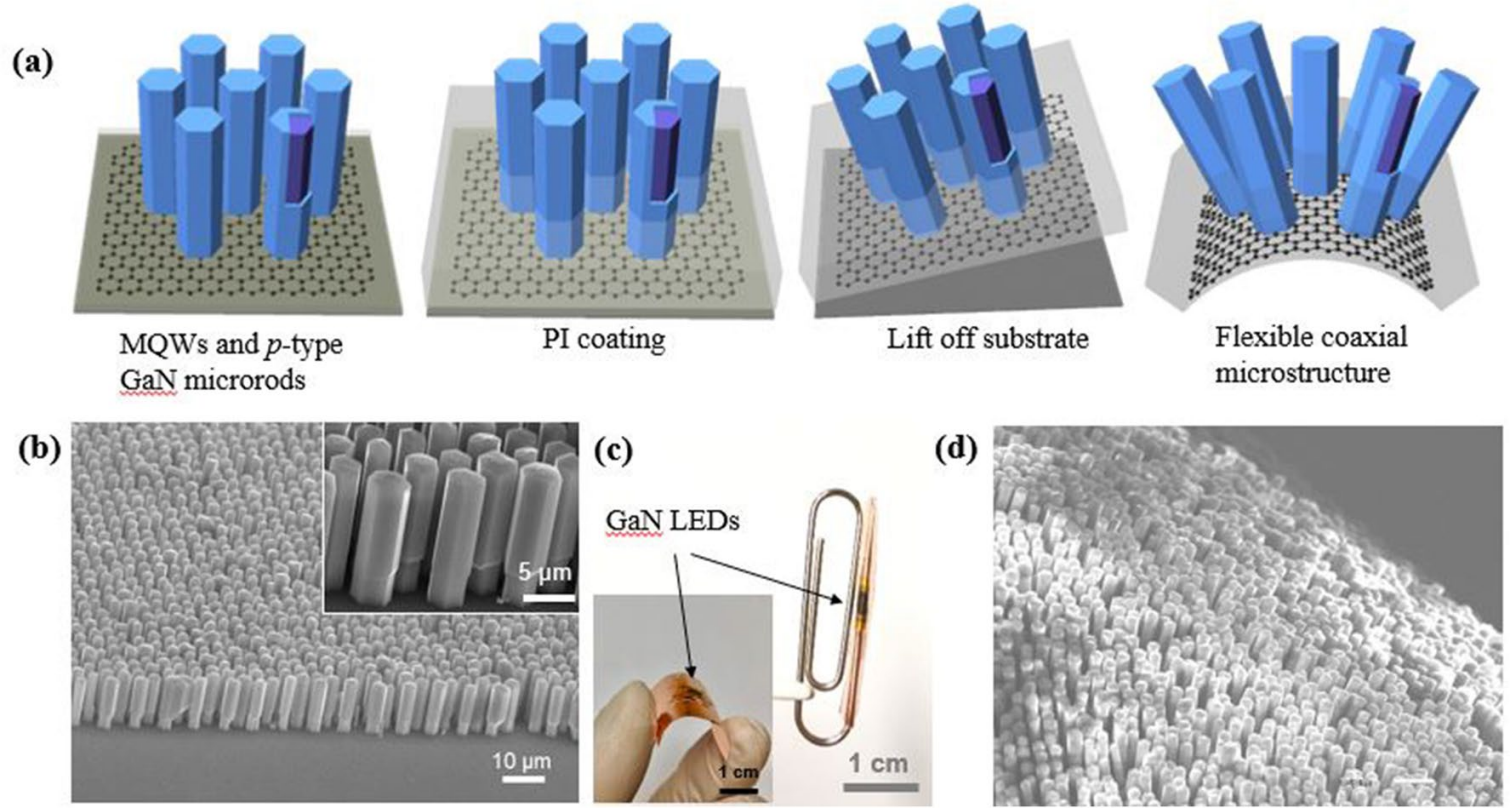
Position-controlled growth of micro-LEDs and flexible properties. (**a**) Schematic illustration of GaN microrods growth on graphene films. (**b**) SEM image of micro-LEDs. (**c**) Optical image of bending LEDs on paper clip. The inset in (**c**) shows the GaN microrods are bent by hand. (**d**) Bent SEM image of micro-LEDs.

The fabrication process of flexible LEDs is illustrated in Fig. 4a. To fabricate coaxial microstructure LEDs, In_x_Ga_1-x_N/GaN multiple-quantum-well structures and *p*-GaN were coaxially grown on the *n*-type GaN microrods^22–25^. Figure 4b shows the SEM images after the growth of the MQWs and *p*-GaN layers. The overgrowth of MQWs and *p*-GaN on the GaN microrods increased the microrod diameter from 4 to 5 μm, indicating that the lateral growth rate was 8 nm/min. After the growth of the coaxial microrod heterostructures, polyimide (PI) was spin-coated on the heterostructures, and a buffered oxide etchant was used to etch the sacrificial SiO_2_ from the 300-nm-thick SiO_2_/Si substrate. The device structure was transferred onto a flexible substrate. The coaxial microrod heterostructures could tolerate the ultimate bending conditions due to the micrometer-sized spacing of the micro-LED arrays. The GaN micro-LEDs were wrapped around a 1-mm-diameter paper clip to test the effect of mechanical deformation, as shown in Fig. 4c, the inset of which shows the micro-LEDs bent manually. Figure 4d shows a SEM image of the GaN microrod LED structures transferred onto the Cu foil in a flexible form; during the bending test, there was no evidence of mechanical damage or fracture.

The flexible LEDs on graphene films were fabricated using the coaxial microrod heterostructures consisting of *p*-GaN, In_x_Ga_1-x_N/GaN MQWs, and *n*-GaN on CVD graphene films. To fabricate flexible micro-LEDs, thin Ni/Au layers (10/10 nm) were deposited on the surface of coaxial *p*-GaN microrods. The micro-LEDs on the graphene films were lifted from the sacrificial substrate and easily transferred onto the Cu foil. Subsequently, the flexible LEDs were transferred onto the Cu foil substrate to form an electrode. The current was injected directly through the graphene, thereby generating a vertical structure for the microstructure LEDs.

**Figure 5:**
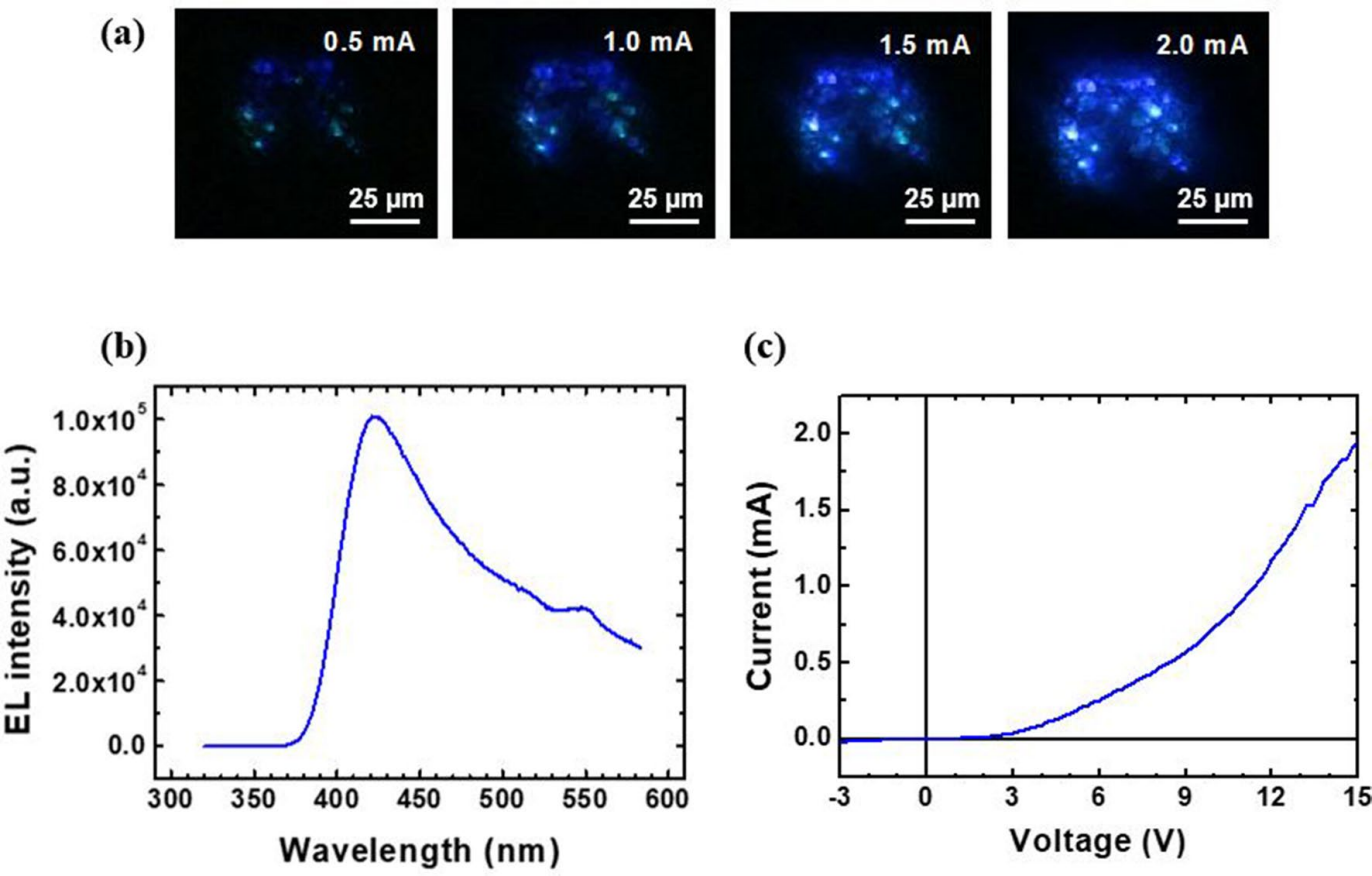
Visible micro-LEDs on graphene. (**a**) Optical microscopy images of light emission from the LED at different applied currents. (**b**) EL spectra from micro-LEDs (**c**) I–V characteristic curve of the LED.

We investigated the light-emitting characteristics of the microstructure LEDs through electroluminescence (EL) spectroscopy. As shown in Fig. 5a, the fabricated 50 × 50 μm^2^ LED devices captured strong blue light emission at applied biases ranging from 0.5 to 2.0 mA. The intensity of emitted light was very strong as visibly seen. It was able to brighten up the room. Figure 5b shows the corresponding room-temperature EL spectra for the LEDs. A peak was measured at 421 nm at an input voltage of approximately 5 V, which could be ascribed to the emission from the GaN/In_x_Ga_1−x_N MQWs embedded in the LEDs. The EL spectra also showed longer wavelength tail next to the 421 nm peak and a small peak near 550 nm. We believe that the longer wavelength EL emissions are related to the variable composition and thickness of In_x_Ga_1-x_N/GaN MQWs coated on different facets of GaN microrods which is typically observed in other multifacetted GaN nanostructured LEDs^26,27^. In addition, the current–voltage (*I–V*) characteristic curve of the LED, as shown in Fig. 5c, indicated the occurrence of rectifying behavior with a turn-on voltage of 3 V and leakage current of 1.5 × 10^−4^ A at –3 V, indicating the formation of a *p*–*n* junction LED.

## Conclusion

We realized the dimension- and position-controlled growth of GaN micro-LEDs on graphene films by using a ZnO seed layer. The use of a ZnO seed layer helped to enhance the nucleation of GaN on graphene and ensured the formation of GaN microrod arrays. The GaN micro-LEDs had a high crystalline structure and high optical quality, as determined by TEM, PL, and EL measurements. Using the dimension- and position-controlled microstructures, micro-LEDs including diverse quantum nanostructures were fabricated on graphene films, leading to the development of high-resolution three-dimensional micro-LEDs and micro devices on graphene films. In particular, we fabricated flexible GaN/In_x_Ga_1−x_N/GaN coaxial quantum nanostructures on graphene films, which exhibited strong blue emission; moreover, the diodes exhibited rectifying behavior. The combination of dimension- and position-controlled GaN quantum nanostructures and 2D graphene films can facilitate the realization of other types of flexible device applications with multi-functional characteristics and novel physical properties.

## Supplementary Information

Supplementary Figures.
