# Supplementary material for: Dimension- and position-controlled growth of GaN microstructure arrays on graphene films for flexible device applications
Source: Sci Rep. 2021 Sep 1;11:17524. doi: 10.1038/s41598-021-97048-2 (PMC8410817; doi:10.1038/s41598-021-97048-2)
Supplement: Supplementary file 1 — Supplementary Figures. [file 41598_2021_97048_MOESM1_ESM.docx]

Supplementary Information

**Dimension- and position-controlled growth of GaN microstructure arrays on Graphene films for flexible device applications**

Dongha Yoo^1^, Keundong Lee^1^, Youngbin Tchoe^1^, Puspendu Guha^2^, Asad Ali^1^, Rajendra K. Saroj^1^, Seokje Lee^1^, A. B. M. Hamidul Islam^1^, Miyoung Kim^2^ and Gyu-Chul Yi^1*^

^1^*Department of Physics and Astronomy, Institute of Applied Physics, and Research Institute of Advanced Materials, Seoul National University, Seoul 151-747, Korea*

^2^*Department of Materials Science and Engineering, Research Institute of Advanced Materials, Seoul National University, Seoul, 151-744, Korea*

*^*^Authors to whom correspondence should be addressed; E-mail:* [*gcyi@snu.ac.kr*](mailto:gcyi@snu.ac.kr)


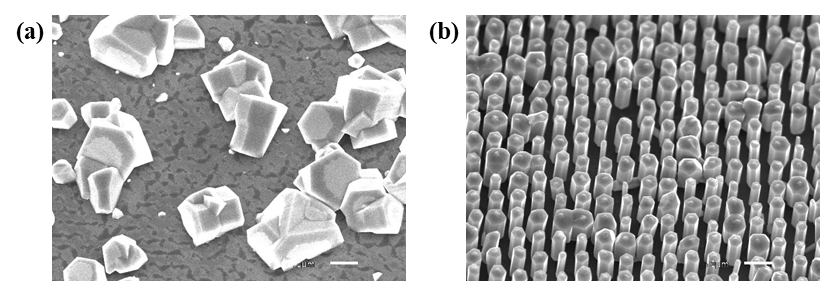


**Figure S1.** Growth of GaN microrods (a) without and (b) with ZnO seed layer

The ZnO seed layer is critical for increasing the nucleation of GaN microstructures on graphene sheets that grow vertically aligned. The primary method we used to achieve controlled growth of GaN microstructures on the graphene surface was to improve nucleation and control the development direction of the microstructures. We used a ZnO seed layer to improve GaN nucleation on graphene since the absence of chemical reactivity of graphene makes it difficult to create dimension-controlled GaN microstructures on graphene films. Only a few micrometer-sized islands grown randomly, as seen in supplemental Figure S1(a), due to the uncommon nucleation of GaN on pristine graphene. Because of the ZnO seed layer, GaN microrods were vertically oriented on graphene films in Figure S1(b).


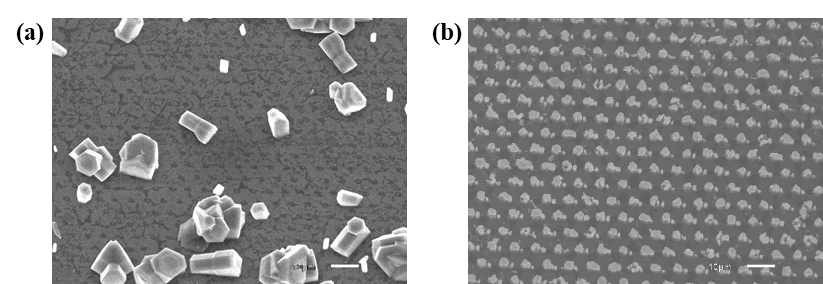


Figure S2. Growth of GaN microrods (a) at 1060–1140 °C without 470 °C, (b) at 470–1140 °C without 1060 °C

Setting appropriate growth settings allowed the GaN microstructures to grow in a position- and dimension-controlled method. First, we employed a temperature-growth approach with multiple steps. A few micrometer-size islands appeared randomly when GaN was grown at a high temperature of 1060–1140 °C without the low-temperature growth step at 470 °C, as shown in Figure S2(a), possibly because the ZnO seed layer was etched off under a hydrogen environment at a high temperature. The GaN could be selectively grown using two-step growth at 470 and 1140 °C, as shown in Figure S2(b). However, at excessively high temperatures, the GaN microstructure could not be epitaxially formed.


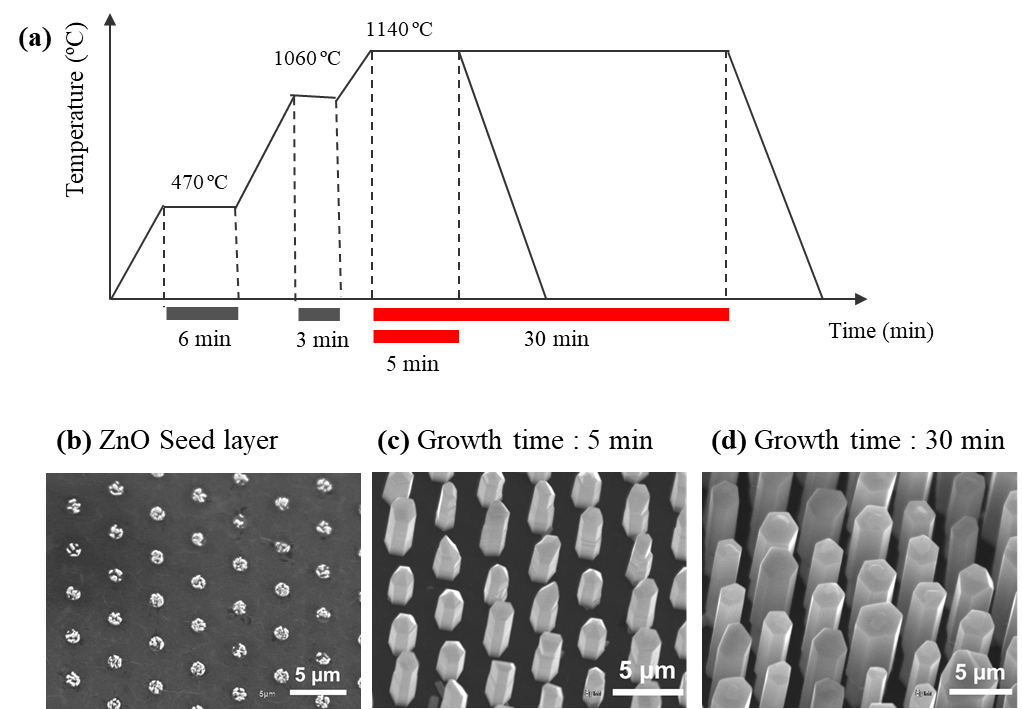


Figure S3. Growth of GaN microrods with different time. (a) Graph of three-step growth method with different growth time, (b) ZnO seed layer, (c) GaN was grown for 5 minutes, (d) GaN was grown for 30 minutes

A growth time method was used to control the height of the GaN microrods. As demonstrated in Figure S3(a), we employed a different growth time method. The SEM image of the ZnO seed layers before GaN development is shown in Figure S3(b). The ZnO seed layers were selectively grown on graphene films. The reactants were oxygen gas (99.9999%) and high purity (Electronic grade) Diethylzinc (DEZn). The bubbler temperature of DEZn was maintained at -10 ^⁰^C and Ar gas (99.9999%) was used as a carrier to carry DEZn from bubbler to the reactor. Oxygen was directly injected in to the reactor during the growth of ZnO seed layers. The flow rates used for both DEZn and oxygen were 20 SCCM and 40 SCCM respectively. The growth was carried out at 600 ^⁰^C for 5 minutes at a reactor pressure of 3.0 Torr. The growth rate of the chamber was 50 nm/min and the height of the ZnO seed layers were approximately 250 nm. SEM images of GaN microstructures grown at varied growth times of 5 minutes and 30 minutes are shown in Figure S3 (c) and (d). The SEM images indicate that diameters and heights of the microstructures were 2.4 $\pm$ 0.2 and 4.0 $\pm$ 0.6, 2.5 $\pm$ 0.3 and 13.0 $\pm$ 0.8 for 5 minutes and 30 minutes, respectively. In particular, with the increase in the growth time, the diameter and height of the microstructures increased.
